# Inhibitory Effect and Potential Mechanism of Trans-2-Hexenal Treatment on Postharvest *Rhizopus* Rot of Peach Fruit

**DOI:** 10.3390/foods14132265

**Published:** 2025-06-26

**Authors:** Xuanyi Cai, Wen Xiang, Liangyi Zhao, Ziao Liu, Ye Li, Yuan Zeng, Xinyan Shen, Yinqiu Bao, Yonghua Zheng, Peng Jin

**Affiliations:** College of Food Science and Technology, Nanjing Agricultural University, Nanjing 210095, China; 2024108017@stu.njau.edu.cn (X.C.); 2023108027@stu.njau.edu.cn (W.X.); t2024063@njau.edu.cn (L.Z.); 2023108026@stu.njau.edu.cn (Z.L.); 9231810709@stu.njau.edu.cn (Y.L.); 2022108017@stu.njau.edu.cn (Y.Z.); 2022108038@stu.njau.edu.cn (X.S.); 2021208011@stu.njau.edu.cn (Y.B.); zhengyh@njau.edu.cn (Y.Z.)

**Keywords:** trans-2-hexenal, *Rhizopus* rot, pathogenesis-related proteins (PRs), phenylpropanoid metabolism, antioxidant defense

## Abstract

Peach fruit faces severe postharvest losses due to thin epidermis and susceptibility to *Rhizopus stolonifer*-induced soft rot. Chemical control risks residue and resistance issues, demanding eco-friendly alternatives. This study elucidated the mechanism by which trans-2-hexenal (E2H) mitigated postharvest soft rot caused by *Rhizopus stolonifer* in peach (*Prunus persica* cv. Hujing Milu) fruit. The results demonstrated that E2H treatment significantly delayed lesion expansion by 44.7% and disease incidence by 23.9% while effectively maintaining fruit quality by delaying firmness loss, reducing juice leakage, and suppressing malondialdehyde (MDA) accumulation. E2H treatment upregulated phenylpropanoid pathway gene expression, enhancing key phenylpropanoid metabolism enzymes activities (phenylalanine ammonia-lyase (PAL), cinnamate 4-hydroxylase (C4H), 4-coumarate-CoA ligase (4CL), polyphenol oxidase (PPO), peroxidase (POD)), leading to the increase of total phenolics by 7.9%. E2H treatment analysis revealed significant enhancements in both chitinolytic activity (CHI) and β-1,3-glucanase (GLU) activity by 85.7% and 12.9%, indicating potentiation of the enzymatic defense system. Concurrently, E2H treatment could improve the redox modulation capacity of peach fruits through promoting catalytic efficiency of redox-regulating enzymes, increasing the accumulation of ascorbic acid (AsA) by 8.1%, inhibiting the synthesis of dehydroascorbic acid (DHA) by 18.6%, as well as suppressing the biosynthesis of reactive oxygen species (ROS). These coordinated enhancements in pathogenesis-related proteins (CHI, GLU), phenylpropanoid metabolism activation, and antioxidant systems are strongly associated with E2H-induced resistance against *Rhizopus stolonifer*, though contributions from other factors may also be involved.

## 1. Introduction

Peach fruit is a drupe of the Rosaceae family, belonging to the genus *Prunus*. Peach fruit is widely favored by consumers due to its vibrant coloration, unique taste, tender and succulent consistency, and abundant nutritional content [1]. The peach, because of its thin skin and soft flesh that is prone to mechanical damage, coupled with the high-temperature harvest season, is very susceptible to pathogenic bacteria infestation and spoilage [2,3]. The predominant fungal pathogens associated with postharvest decay in peaches are *Monilinia fructicola* [4,5,6], *Rhizopus stolonifer* [7], and *Botrytis cinerea* [8]. *Rhizopus stolonifer*, a fungal species classified within the subphylum Zygomycota, is a prominent causal agent of postharvest *Rhizopus* rot in peaches. This pathogen is recognized as one of the most devastating postharvest pathogens of peach fruit, significantly compromising their storage quality and commercial value. This pathogen directly contributes to 10–40% of postharvest losses in peach fruit depending on environmental conditions and handling practices. Consequently, it is regarded as one of the primary contributors to postharvest losses in peach production [9,10]. Given its substantial impact, research on the postharvest diseases of peach fruit has garnered significant attention and has emerged as a prominent topic in the domain of fruit and vegetable preservation studies.

The current methods of postharvest fruit disease control are mainly classified as physical, chemical, biological and composite methods [11,12]. Currently, chemical fungicides, including Carbendazim, Thiophanate-methyl, and Azoxystrobin, are widely employed for the management of postharvest diseases in peach fruit [13]. Chemical fungicides are widely recognized for their benefits, including broad-spectrum efficacy, cost-effectiveness, and operational convenience. Nevertheless, their prolonged use has led to significant concerns regarding chemical residue accumulation and the emergence of resistance, which pose serious threats to environmental integrity and human health [14]. Given this, it is crucial to focus on developing environmentally sustainable and safer antimicrobial strategies as viable alternatives to conventional chemical fungicides [15]. Among these alternatives, plant extracts have gained significant attention as a promising research focus owing to their inherent safety, multifaceted mechanisms of action, and high environmental compatibility [16,17].

Trans-2-hexenal (E2H) is a naturally occurring volatile organic compound found in numerous plant species, such as apples, peaches, and strawberries [18], and is a plant-based extract recognized for its potent antifungal activity, safety profile, and environmental sustainability. It has been extensively employed as a fumigant to effectively suppress the growth of postharvest pathogenic fungi [19,20]. Extensive research has demonstrated that E2H exhibits significant inhibitory effects against a broad spectrum of postharvest pathogenic fungi. Specifically, E2H effectively suppressed the mycelial growth of *Penicillium digitatum*, decreasing the severity of green mold in citrus [21]; inhibited both sporulation and patulin biosynthesis in *Penicillium expansum*, resulting in a reduction in kiwifruit decay during storage [19]; and completely suppressed the growth of *Aspergillus flavus*, achieving 100% inhibition of peanut seed contamination at a low fumigation concentration of 0.05 μL/L [20]. E2H, as a natural plant-derived compound, has emerged as a promising alternative in postharvest management due to its non-toxic nature, potent antimicrobial activity, minimal residue accumulation, and excellent environmental compatibility. These advantageous properties have facilitated its extensive utilization in both in vitro antifungal studies and practical applications for prolonging the storage period of diverse fresh produce. Despite its promising potential, research on its effectiveness in preventing postharvest infections in peaches remains unexplored.

Chitinase (CHI) and β-1,3-glucanase (GLU), two key pathogenesis-related (PR) proteins, play critical roles in plant defense by enzymatically degrading chitin and β-1,3-glucan, the critical structural elements of fungal cell walls [22,23,24]. This degradation process effectively restricts hyphal extension and limits further pathogen invasion. The phenylpropanoid metabolism pathway represents a crucial biosynthetic route in plants and is responsible for biosynthesizing diverse secondary metabolites, like phenylpropanoid derivatives, lignin, and phytoalexins, all of which demonstrate significant antimicrobial activity [25,26]. Research has shown that Agaro-oligosaccharides improve the defense responses of peach fruit against phytopathogens by increasing CHI and GLU activities, alongside elevating enzymatic activities and gene expression levels of phenylpropanoid metabolism components, resulting in an increase in total phenolics, flavonoids, and lignin content [27]. Similarly, postharvest exogenous MeJA treatment in kiwifruit upregulated the activities and transcriptional activities of defense-associated enzymes, such as PAL, PPO, and CHI, significantly improving resistance against *Botryosphaeria dothidea* [28]. In strawberry fruit, natamycin and potassium sorbate treatment stimulated phenylpropanoid pathway activity as well as pathogenesis-related protein activities (CHI and GLU), promoting the increase in total phenolics, flavonoids, and lignin content, thereby enhancing resistance to *Botrytis cinerea* infection [29]. These findings highlight the critical importance of promoting phenylpropanoid metabolism and increasing the functionality of CHI and GLU in fortifying disease resistance mechanisms in peach fruit.

After being infected by pathogens, plants undergo an oxidative burst [30]. The swift buildup of reactive oxygen species (ROS) in plants results in the decay of cellular membranes, induces oxidative damage to intracellular DNA, disrupts normal metabolic processes, diminishes the disease resistance of plant tissues, and accelerates both tissue senescence and the onset of diseases [31]. Therefore, maintaining the balance of ROS is crucial for the integrity and functionality of plant tissues. Yang et al. reported that MeJA and MeSA treatment markedly promoted the catalytic efficiency of redox-regulating enzymes (superoxide dismutase (SOD), catalase (CAT), ascorbate peroxidase (APX), glutathione reductase (GR)) and elevated antioxidant compounds contents (ascorbic acid (AsA) and glutathione (GSH)) in Chinese winter jujube, thereby maintaining redox homeostasis and delaying the onset of black rot caused by *Alternaria tenuissima* [32]. Similarly, treatment with γ-aminobutyric acid (GABA) upregulated the functionality and transcription levels of antioxidant enzymes (SOD, CAT, APX, and GR) in red pitaya fruit, reducing ROS accumulation and improving resistance against *Gilbertella persicaria* [33]. In parallel, application of the biocontrol strain HG03 balanced ROS metabolism in peach fruit, strengthened free radical scavenging capacity and significantly improved disease resistance [34]. The above studies indicate that maintaining the balance of ROS is essential for increasing resistance to diseases in peaches.

The purpose of this research was to evaluate the impact of E2H treatment on fruit quality and postharvest *Rhizopus* rot control in peach fruit. By systematically analyzing the dynamics of PR proteins, phenylpropanoid metabolism, and ROS metabolism, we sought to elucidate the underlying mechanisms through which E2H enhances disease resistance. The findings were expected to provide a theoretical foundation for subsequent molecular-level investigations on the biocontrol potential of plant-derived volatiles in postharvest management systems.

## 2. Materials and Methods

### 2.1. Fruit Materials and Treatment

The fungal strain *Rhizopus stolonifer* (Ehrenb. ex Fr.) Vuill. was subcultured three times on potato dextrose agar (PDA) medium at 26 ± 0.5 °C (85 ± 2% RH). Spores were harvested aseptically, filtered, and adjusted to 1 × 10^5^ spores/mL using a hemocytometer.

Peach fruits (*Prunus persica* cv. Hujing Milu) harvested at commercial maturity from Jiangsu Academy of Agricultural Sciences in July 2024 were selected for uniformity. Following a completely randomized design, fruits were surface-sterilized with 0.1% NaClO (2 min), rinsed, dried, and randomly assigned to two experimental groups (*n* = 60 per group) using random number generation: control group and E2H treatment group (2 µL/L fumigation, based on preliminary results). Following fumigation, two sterile wounds (3 mm diameter × depth) per fruit were inoculated with 15 μL of *R. stolonifer* suspension (1 × 10^5^ spores/mL), then stored at 20 ± 1 °C with 90–95% RH (maintained using a constant temperature and humidity incubator). The 60 h observation period was chosen because the pre-experiment confirmed that the control group completed the entire disease process within this time and the key effect window period of E2H treatment covered this interval. Quality parameters (firmness, total soluble solids (TSS), and juice yield) and infection progression (0–60 h post-inoculation) were monitored. Disease incidence and lesion diameters were recorded at 12 h intervals (24–60 h). Lesion border tissues (2–3 mm transition zone between healthy and infected tissues) were aseptically excised with sterile blades, flash-frozen in liquid nitrogen, and stored at −80 °C for biochemical analysis.

### 2.2. Disease Incidence and Lesion Diameter Assessment

Lesion diameter was quantified through perpendicular diameter measurement, where two measurements were taken at right angles across the lesion and averaged. Infection was defined as lesions exceeding 3 mm (confirmed under standardized lighting conditions). The incidence of disease was determined by the ratio of infected wounds compared to all inoculated wounds.

### 2.3. Measurement of Firmness, Yield of Juice, and Total Soluble Solids (TSS)

Fruit firmness was measured with a GY-3 fruit firmness tester (Top Cloud-agri, Hangzhou, China) equipped with a 5 mm diameter probe. Five measurements per fruit (three biological replicates per treatment) were recorded. Results were expressed in Newtons (N).

The yield of the juice was determined using a centrifugal method [35]. Cylindrical tissue samples (10 mm diameter × 5 mm height) were obtained from three fruits per treatment using a cork borer, placed in pre-weighed tubes (M1) with absorbent paper, weighed (M2), and centrifuged (2000× *g*, 10 min). Following centrifugal processing, the tissue was removed and then reweighed (M3). The yield of the juice was determined by applying the following formula:

(1) Yield of juice (%) = [(M3 − M1)/(M2 − M1)] × 100%

Fruit juice was extracted by homogenizing and filtering the tissue samples. TSS was measured using a refractometer (Model 14081S, Atago Co., Tokyo, Japan) calibrated with deionized water. All experimental samples underwent triplicate technical repetitions and the results were expressed as a percentage (%).

### 2.4. Determination of Malondialdehyde (MDA) Content and Total Phenolic Content

MDA content was determined by the thiobarbituric acid (TBA) method [35]. One gram of tissue was homogenized in 5 mL 10% trichloroacetic acid (TCA) solution and centrifuged at 10,000× *g* for 20 min at 4 °C. The supernatant (1.5 mL) was reacted with an equal volume of 0.67% TBA solution at 95 °C for 30 min (controls were prepared by substituting TCA solution for supernatant). Absorbance was measured at 450 nm, 532 nm, and 600 nm.

Total phenolic content was quantified using the Folin–Ciocalteu assay [36]. One gram of tissue was homogenized in 5 mL ice-cold 80% acetone and centrifuged at 12,000× *g* for 20 min at 4 °C. The supernatant (40 μL) was reacted with 160 μL H_2_O, 1 mL Folin–Ciocalteu reagent, and 0.8 mL 7.5% Na_2_CO_3_ solution in darkness at 30 °C for 1 h. Absorbance at 765 nm was measured.

### 2.5. Determination of Chitinase (CHI) and β-1,3-Glucanase (GLU) Activities

CHI activity was assayed by the method of Abeles et al. [24] with modifications. One gram of tissue was homogenized in 6 mL ice-cold extraction buffer. The homogenate was centrifuged at 12,000× *g* for 20 min at 4 °C; the supernatant was collected as the enzyme source. The reaction system (total volume 2 mL) contained 1.2 mL enzyme extract and 0.7 mL 1% colloidal chitin. After incubation at 40 °C for 1 h, a 0.5 mL aliquot was mixed with 0.1 mL 7.5% Na_2_CO_3_, boiled for 10 min, and ice-cooled. This mixture was then reacted with p-dimethylaminobenzaldehyde (DMAB) (2 mL) at 37 °C for 40 min. Absorbance at 524 nm was measured spectrophotometrically.

GLU activity was assayed as follows: One gram of tissues was homogenized in 5 mL extraction buffer and centrifuged at 12,000× *g* for 20 min at 4 °C. The reaction system consisted of 200 μL enzyme extract and 100 μL 0.4% laminarin. After incubation at 37 °C for 40 min, the reaction was terminated by adding 1.7 mL H_2_O and 1.5 mL 3,5-dinitrosalicylic acid reagent. The mixture was boiled for 3 min, cooled to room temperature, and diluted to a final volume of 25 mL with distilled water. Absorbance at 524 nm was measured.

### 2.6. Assays of Phenylpropanoid Metabolism-Related Enzyme Activities

PAL activity was assayed as follows: One gram of tissue was homogenized in 5 mL extraction buffer and centrifuged at 12,000× *g* for 30 min at 4 °C. The reaction system contained 3 mL borate buffer, 0.5 mL 20 mM L-phenylalanine, and 0.5 mL enzyme extract (control: heat-inactivated enzyme boiled for 5 min). After incubation at 37 °C for 1 h, the reaction was terminated with 0.1 mL 6 M HCl. Absorbance at 290 nm was measured.

C4H activity was assayed by the method of Zhang et al. [37]. One gram of tissue was homogenized in 5 mL 50 mM Tris-HCl (pH 7.5) and centrifuged at 12,000× *g* for 20 min at 4 °C. The reaction system consisted of 0.5 mL enzyme extract in Tris–HCl. After incubation at 25 °C for 30 min, absorbance at 340 nm was measured.

4CL activity was assayed by the method of Zhang et al. [37], with modifications. The reaction system (in 50 mM Tris–HCl, pH 7.5) contained 0.5 mL enzyme extract, 2 mL 5 mM MgCl_2_, 0.5 mL 5 mM ATP, 0.05 mL 0.6 mM p-coumarate, and 0.05 mL 0.4 mM CoA. After incubation at 40 °C for 15 min, absorbance at 333 nm was measured.

PPO activity was assayed by the method of Zhang et al. [37]. The reaction system contained 4 mL 50 mM sodium acetate buffer, 1 mL 50 mM catechol, and 100 μL enzyme extract. Absorbance at 420 nm was measured at 30 s intervals for 3 min.

POD activity was determined according to Zhang et al. [37]. The reaction system consisted of 3 mL 25 mM guaiacol, 0.2 mL 0.5 M hydrogen peroxide (H_2_O_2_), and 0.5 mL enzyme extract in sodium acetate buffer. Absorbance at 470 nm was measured at 30 s intervals for 3 min.

### 2.7. Determination of ROS Parameters

DPPH radical scavenging capacity was assayed by the method of Zhu et al. [38]. One gram of tissue was homogenized in 5 mL 50% ethanol and centrifuged at 12,000× *g* for 20 min at 4 °C. The reaction mixture contained 0.1 mL supernatant and 1.9 mL 120 μM DPPH ethanol solution. After incubation at 25 ± 0.5 °C in darkness for 30 min, absorbance at 525 nm was measured.

Hydroxyl radical (•OH) scavenging capacity was determined according to Hou et al. [39]. The reaction system consisted of 0.3 mL supernatant, 1.5 mL 18 mM salicylate–ethanol solution, 2 mL 18 mM FeSO_4_ solution, and 0.1 mL 0.3% H_2_O_2_. Following incubation at 37 ± 0.5 °C in darkness for 30 min, absorbance at 510 nm was recorded.

The superoxide anion (O_2_^•−^) production rate was assayed by the method of Zuo et al. [40]. One gram of tissue was homogenized in 5 mL sodium phosphate buffer (50 mM, pH 7.8) and centrifuged at 12,000× *g* for 20 min at 4 °C. The reaction system comprised 1 mL homogenate, 1 mL 1 mM hydroxylamine hydrochloride (NH_2_OH·HCl), and 1 mL buffer. After incubation at 25 ± 0.5 °C for 1 h, 1 mL 17 mM sulfanilic acid and 1 mL 7 mM α-naphthylamine were added, followed by additional incubation for 20 min. Absorbance at 530 nm was measured.

H_2_O_2_ content was assayed by the method of Alexieva et al. [41]. One gram of tissue was homogenized in 5 mL ice-cold acetone and centrifuged at 12,000× *g* for 20 min at 4 °C. The supernatant (1 mL) was reacted with TiCl_4_-HCl/NH_3_. The mixture was centrifuged at 12,000× *g* for 15 min. The precipitate was washed twice with ice-cold acetone and dissolved in 3 mL 2 M H_2_SO_4_. Absorbance at 412 nm was measured.

### 2.8. Assays of Superoxide Dismutase (SOD) and Catalase (CAT) Activities

SOD activity was assayed by the method of Ma et al. [42], with modifications. The reaction system (total volume 3.1 mL) contained 0.1 mL enzyme extract, 1.8 mL sodium phosphate buffer (50 mM, pH 7.8), 0.3 mL 130 mM methionine, 0.3 mL 750 μM nitroblue tetrazolium, 0.3 mL 100 μM EDTA, and 0.3 mL 20 μM riboflavin. After exposure to 4000 lux light at 25 ± 0.5 °C for 15 min, absorbance at 560 nm was measured.

CAT activity was determined according to Zhang et al. [43]. One gram of tissue was homogenized in 5 mL sodium phosphate buffer (50 mM, pH 7.0) and centrifuged at 12,000× *g* for 20 min at 4 °C. The reaction system (total volume 3.0 mL) consisted of 0.2 mL enzyme extract, 2.5 mL 50 mM sodium phosphate buffer, and 0.3 mL 0.75% H_2_O_2_. The decrease in absorbance at 240 nm was monitored spectrophotometrically at 30 s intervals for 3 min.

### 2.9. Determination of Ascorbate-Glutathione (AsA-GSH) Cycle-Related Parameters

Frozen tissue (1.0 g) was homogenized in 5 mL ice-cold sodium phosphate buffer and centrifuged at 12,000× *g* for 20 min at 4 °C. The supernatant was collected for enzymatic analyses.

Monodehydroascorbate reductase (MDHAR) and dehydroascorbate reductase (DHAR) activities were assayed by the method of Chumyam et al. [44], with modifications:

MDHAR activity: The reaction mixture (total volume 2.0 mL) contained 1.5 mL sodium phosphate buffer (pH 7.0), 0.4 mL enzyme extract, and 0.1 mL 0.25 U ascorbate oxidase. The decrease in absorbance at 340 nm was monitored at 30 s intervals for 3 min.

DHAR activity: The reaction system (3.0 mL) consisted of 2.8 mL sodium phosphate buffer (pH 7.0), 0.1 mL enzyme extract, and 0.1 mL 2 mM dehydroascorbic acid (DHA). The increase in absorbance at 265 nm was tracked at 30 s intervals for 3 min.

APX and GR activities were determined according to Wang et al. [45], with modifications:

APX activity: The reaction mixture (3.0 mL) contained 2.6 mL sodium phosphate buffer (pH 7.5), 0.3 mL 2 mM H_2_O_2_, and 0.1 mL enzyme extract. The decline in absorbance at 290 nm was recorded.

GR activity: The reaction system (3.0 mL) comprised 2.7 mL sodium phosphate buffer (pH 7.5), 0.1 mL 5 mM oxidized glutathione (GSSG), 0.2 mL enzyme extract, and 40 μL 4 mM NADPH. The decrease in absorbance at 340 nm was monitored.

AsA and DHA contents were quantified following Chumyam et al. [44]. One gram of tissue was homogenized in 5 mL 6% TCA solution and centrifuged at 12,000× *g* for 20 min at 4 °C. The supernatant was analyzed spectrophotometrically at 265 nm.

### 2.10. Differential Gene Expression Analysis

We commissioned GENE DENOVO (Guangzhou, China) to perform RNA-Seq sequencing and analysis. Raw reads were quality-controlled using fastp to filter out low-quality data and obtain clean reads. HISAT2 was used to align the clean reads to the peach reference genome (https://www.ncbi.nlm.nih.gov/genome/?term=prunus+persica (accessed on 12 September 2024)), to determine the distribution and annotation of reads across different intervals. Stringtie was used to reconstruct transcripts, and RSEM was employed to calculate the expression levels of all genes in each sample. Differential gene expression profiles were analyzed via DESeq2 (v1.40.2), implemented in the R statistical framework (v4.3.1). The raw read counts from gene expression quantification were processed through a three-step analytical pipeline: median-of-ratios normalization (sequencing depth adjustment), negative binomial generalized linear model (GLM)-based *p*-value calculation, and Benjamini–Hochberg false discovery rate (FDR) correction.

### 2.11. Data Processing and Statistical Analysis

Experiments were conducted with triplicate biological replicates across two independent trials. The data processing was conducted using Microsoft Excel 2021 and IBM SPSS v26, while graphical outputs and correlation matrices were generated via Origin 2021. Statistical analyses employed one-way ANOVA with Student’s t-test (SPSS v26). The significance threshold was *p* < 0.05.

## 3. Results

### 3.1. Effects of E2H Treatment on Postharvest Rhizopus Rot Development and Fruit Quality in Peach

**Figure 1:**
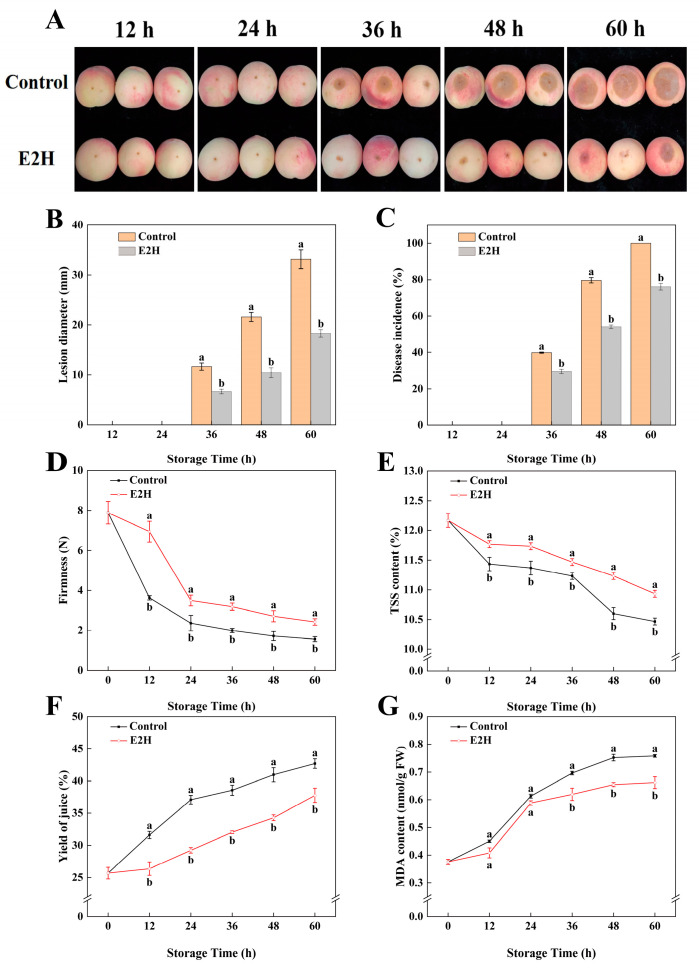
Effects of E2H treatment on: (**A**) visual appearance; (**B**) lesion diameter; (**C**) disease incidence; (**D**) firmness; (**E**) total soluble solids (TSS) content; (**F**) yield of juice; and (**G**) malondialdehyde (MDA) content in postharvest peach fruit stored at 20 ± 1 °C with 90–95% RH. Data points represent sampling intervals at 0, 12, 24, 36, 48, and 60 h post treatment. Values are expressed as mean ± standard deviation (SD) (*n* = 3). Distinct lowercase letters denote significant intergroup differences (*p* < 0.05, Student’s *t*-test).

Throughout the storage period, E2H treatment demonstrated significant inhibitory effects on *Rhizopus* rot development in peach fruit (Figure 1A–C). As shown in Figure 1A, disease symptoms appeared at 36 h post inoculation within the control as well as the E2H-treated group, with smaller lesion diameters observed in the E2H-treated fruits. Following inoculation with *Rhizopus stolonifer*, the control group exhibited a lesion diameter and disease incidence of 11.653 mm and 39.767%, respectively, while the E2H-treated fruits showed significantly lower values of 6.707 mm and 29.545%. At 60 h, control fruits were completely decayed, with 100% disease incidence and a lesion diameter reaching 33.134 mm (Figure 1B,C). In contrast, the E2H-treated fruits maintained a significantly lower disease incidence (76.042%) and lesion diameter (18.324 mm) compared to the control (Figure 1B,C).

As illustrated in Figure 1D, fruit firmness declined progressively during storage; however, E2H treatment effectively delayed this decline. At 12, 24, 36, 48, and 60 h, the firmness of control fruits was only 52.3%, 67.6%, 62.5%, 63.4%, and 64.3% of that observed in the E2H-treated fruits, respectively. Similarly, E2H treatment significantly slowed the decrease in TSS content (Figure 1E).

Juice yield and MDA content exhibited consistent trends, showing continuous increases throughout storage (Figure 1F,G). From 0 to 60 h, the yield of juice increased progressively; however, the E2H-treated fruits consistently showed lower values than the control. The largest difference (21.2%) was observed at 24 h (Figure 1F). Similarly, MDA content in E2H-treated fruits was 87.0% of that in the control at 48 h, representing the maximum difference between the two groups (Figure 1G).

### 3.2. Effects of E2H Treatment on CHI and GLU Activities in Postharvest Peach Fruit

**Figure 2:**
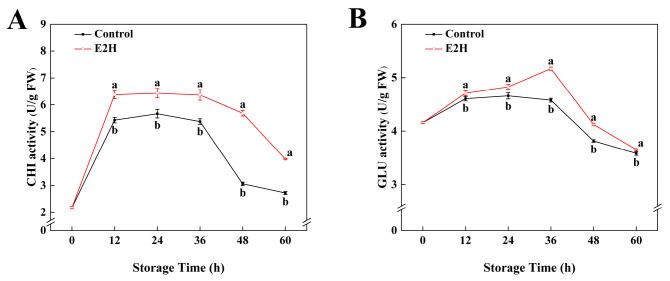
Effects of E2H treatment on enzymatic activities of: (**A**) chitinase (CHI); and (**B**) β-1,3-glucanase (GLU) in postharvest peach fruit stored at 20 ± 1 °C with 90–95% RH. Data points represent sampling intervals at 0, 12, 24, 36, 48, and 60 h post treatment. Values are expressed as mean ± standard deviation (SD) (*n* = 3). Distinct lowercase letters denote significant intergroup differences (*p* < 0.05, Student’s *t*-test).

As shown in Figure 2, CHI and GLU activities exhibited a biphasic pattern characterized by an initial increase followed by a decline during storage. E2H treatment significantly enhanced both enzyme activities in relation to the untreated control. The maximum difference in CHI activity was recorded at 48 h post inoculation, with E2H-treated fruits showing an 85.7% increase over the control (Figure 2A). For GLU activity, the E2H-treated group reached its peak value of 5.17 U/g at 36 h, representing the largest divergence between groups, where the control group retained only 88.5% of the activity observed in the E2H-treated group (Figure 2B).

### 3.3. Effects of E2H Treatment on Phenylpropanoid Metabolism in Peach Fruit

**Figure 3:**
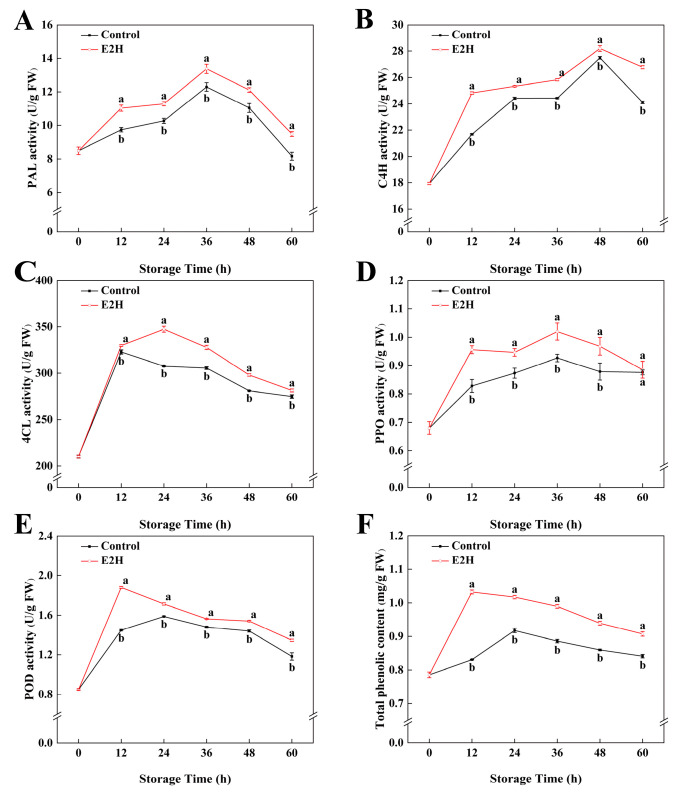
Effects of E2H treatment on enzymatic activities of: (**A**) phenylalanine ammonia-lyase (PAL); (**B**) cinnamate 4-hydroxylase (C4H); (**C**) 4-coumarate-CoA ligase (4CL); (**D**) polyphenol oxidase (PPO); (**E**) peroxidase (POD); and (**F**) total phenolic content in postharvest peach fruit stored at 20 ± 1 °C with 90–95% RH. Data points represent sampling intervals at 0, 12, 24, 36, 48, and 60 h post treatment. Values are expressed as mean ± standard deviation (SD) (*n* = 3). Distinct lowercase letters denote significant intergroup differences (*p* < 0.05, Student’s *t*-test).

As shown in Figure 3A, PAL activity in peach fruits peaked at 48 h post inoculation and subsequently declined gradually. E2H-treated peaches exhibited substantially greater PAL activity compared to the untreated control throughout storage (*p* < 0.05). E2H treatment markedly enhanced C4H activity, with treated peaches exhibiting higher activity than the untreated control throughout all intervals (Figure 3B). The maximum difference occurred at 12 h, when the control group exhibited only 87.3% of the C4H activity observed in the E2H-treated group. The 4CL activity increased rapidly at 12 h and stabilized thereafter (Figure 3C). E2H-treated peaches consistently displayed higher 4CL activity in relation to the untreated control throughout the full storage duration.

POD and PPO activities followed biphasic trends, peaking before declining (Figure 3D,E). E2H treatment significantly increased both POD and PPO activities, with treated peaches maintaining higher levels than the untreated control at all stages. The total phenolic content showed an upward trend initially but later decreased during storage (Figure 3F). E2H treatment promoted phenolic accumulation, resulting in 24.3%, 10.9%, 11.7%, 9.3%, and 7.9% higher total phenolics in treated fruits compared to the control at 12, 24, 36, 48, and 60 h, respectively.

### 3.4. Effects of E2H Treatment on ROS Metabolic Pathways

**Figure 4:**
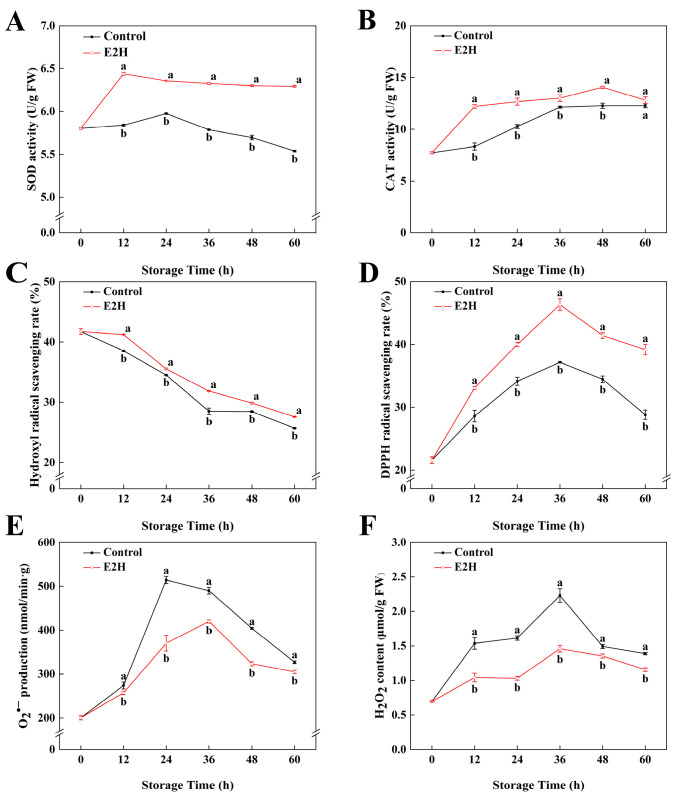
Effects of E2H treatment on: (**A**) superoxide dismutase (SOD) activity; (**B**) catalase (CAT) activity; (**C**) hydroxyl radical (•OH) scavenging capacity; (**D**) DPPH radical scavenging capacity; (**E**) superoxide anion (O_2_^•−^) generation rate; and (**F**) hydrogen peroxide (H_2_O_2_) content in postharvest peach fruit stored at 20 ± 1 °C with 90–95% RH. Data points represent sampling intervals at 0, 12, 24, 36, 48, and 60 h post treatment. Values are expressed as mean ± standard deviation (SD) (*n* = 3). Distinct lowercase letters denote significant intergroup differences (*p* < 0.05, Student’s *t*-test).

As shown in Figure 4A,B, the enzymatic activities of SOD as well as CAT in peach fruits exhibited an initial increase followed by a subsequent decline. E2H treatment induced an elevation in both SOD and CAT activity levels. At 60 h of storage, E2H treatment induced a 0.14-fold elevation in SOD activity relative to the untreated control (Figure 4A). In Figure 4B, E2H treatment significantly enhanced CAT activity in the fruit, with the maximum difference observed at 12 h, where the untreated control exhibited only 68.3% of the CAT activity measured in the E2H-treated group.

In Figure 4C, it can be concluded that the •OH scavenging rate in peach fruits gradually decreased as the storage time increased. E2H treatment effectively delayed the decline in the •OH scavenging rate, with the E2H-treated group showing 1.15-, 1.17-, 1.31-, 1.09-, and 1.43-fold •OH scavenging rates compared to the control group at 12, 24, 36, 48, and 60 h of storage, respectively. The DPPH free radical scavenging rate followed a trend of initial increase followed by decline during storage. E2H treatment significantly enhanced the ability of the peaches to scavenge DPPH radicals (*p* < 0.05), which persisted at a higher level in the E2H-treated group relative to the untreated control during the entire storage time. Production rate of O_2_^•−^ exhibited a similar trend to the DPPH free radical scavenging rate, showing an initial increase followed by a decline. E2H treatment effectively suppressed the increase in O_2_^•−^ production (*p* < 0.05) rate in peach fruits, resulting in the E2H-treated group exhibiting consistently lower O_2_^•−^ production rates relative to the untreated control. As shown in Figure 4F, the H_2_O_2_ content in peach fruits stored at 20 °C exhibited a temporary increase, peaking at 36 h and then decreased steadily. Fruit undergoing pathogen infection exhibits stress responses; ROS bursts are one of the most typical reactions. Consequently, ROS levels rapidly accumulate upon bacterial inoculation. As shown in Figure 4F, the E2H-treated fruits exhibited significantly reduced H_2_O_2_ content, with the E2H-treated group displaying a 34.5% lower H_2_O_2_ content than the untreated control at the peak time point.

### 3.5. Effects of E2H Treatment on AsA-GSH Cycle

**Figure 5:**
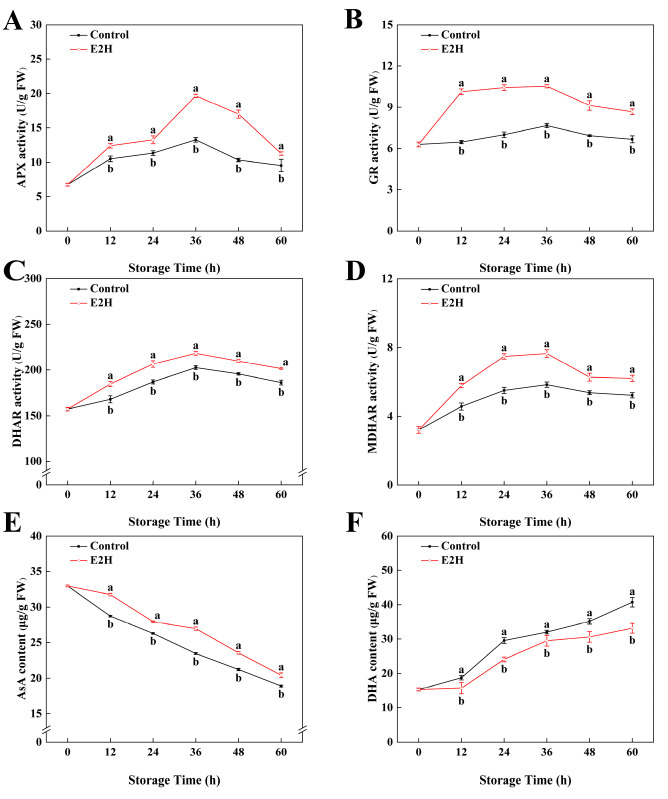
Effects of E2H treatment on enzymatic activities of: (**A**) ascorbate peroxidase (APX); (**B**) glutathione reductase (GR); (**C**) dehydroascorbate reductase (DHAR); (**D**) monodehydroascorbate reductase (MDHAR),; (**E**) ascorbic acid (AsA) content; and (**F**) dehydroascorbic acid (DHA) content in postharvest peach fruit stored at 20 ± 1 °C with 90–95% RH. Data points represent sampling intervals at 0, 12, 24, 36, 48, and 60 h post treatment. Values are expressed as mean ± standard deviation (SD) (*n* = 3). Distinct lowercase letters denote significant intergroup differences (*p* < 0.05, Student’s *t*-test).

As shown in Figure 5A, the activity of APX in peach fruits reached its peak at 36 h of storage and then decreased rapidly afterward. E2H treatment caused an increase in APX activity expression during the early storage period and delayed its decline in the later stages. The maximum difference between the two groups was observed at 36 h, where the APX activity in the E2H-treated group was a 0.48-fold greater compared to the untreated control. Throughout the storage period, GR activity levels remained relatively stable, with the E2H-treated group consistently showing higher GR activity compared to the untreated control (*p* < 0.05). From Figure 5C,D, it can be seen that the activities of DHAR and MDHAR in peach fruits both exhibited a trend of initial increase followed by decline. E2H treatment induced a sustained enhancement in DHAR activity relative to the untreated control. Additionally, E2H treatment enhanced the increase in MDHAR activity; at 60 h of storage, E2H treatment elevated MDHAR activity by 18.8% relative to the untreated control (Figure 5D).

As shown in Figure 5E, the AsA content in peach fruits during storage exhibited a decreasing trend. E2H treatment suppressed the decline in AsA content throughout the entire storage period. In Figure 5F, DHA content in peach fruits gradually increased with extended storage time. The E2H-treated fruits showed a delayed rise in DHA content; at 60 h of storage, the DHA content in the E2H-treated group was 81.4% of that in the untreated control.

### 3.6. Effects of E2H Treatment on Differential Gene Expression in ROS and Phenylpropanoid Metabolism Pathways and Correlation Analysis

**Figure 6:**
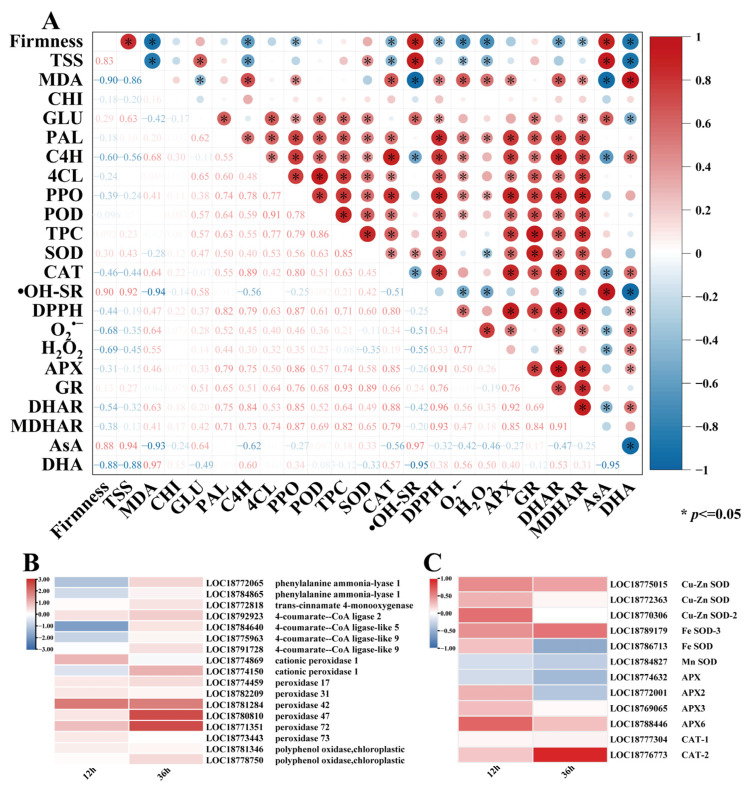
(**A**) Correlation analysis of E2H treatment effects on peach fruit disease resistance (positive and negative correlations are denoted by red and blue hues, respectively, *p* <= 0.05); (**B**) differential gene expression patterns in phenylpropanoid metabolism-related pathways following E2H treatment; and (**C**) differential gene expression patterns in reactive oxygen species (ROS) metabolism-related pathways following E2H treatment.

Correlation analyses of quality indicators, ROS metabolism, phenylpropanoid metabolism, and the AsA-GSH cycle related to peach fruit disease are shown in Figure 6A. Pearson’s coefficients demonstrated statistically significant intervariable correlations (*p* < 0.05) among the postharvest quality and physiological parameters in peach fruit. Fruit firmness showed negative correlations with TSS and MDA content (*p* < 0.05), indicating that texture softening was associated with sugar accumulation and oxidative damage. Within the antioxidant system, SOD and CAT activities exhibited statistically significant positive correlations (*p* < 0.05), as well as APX and GR, demonstrating their synergistic role in maintaining redox homeostasis. Furthermore, oxidative stress markers (MDA, H_2_O_2_, and O_2_^•−^) exhibited negative correlations (*p* < 0.05) with antioxidant capacity (hydroxyl radical scavenging rate (•OH-SR) and SOD activity), confirming the antagonistic relationship between oxidative damage and defense systems. In phenylpropanoid metabolism, key enzymes (PAL, C4H, 4CL, PPO, and POD) showed strong positive correlations (*p* < 0.05) with each other and with the total phenolic content (TPC), suggesting that metabolism activation promoted the accumulation of antimicrobial compounds. Collectively, these data indicate that E2H treatment suppressed *Rhizopus stolonifer* proliferation through coordinately regulating ROS scavenging, phenylpropanoid metabolism, and oxidative balance in peach fruit.

The correlation analysis demonstrated that E2H treatment enhanced disease resistance in peach fruit through coordinated regulation of phenylpropanoid and ROS metabolism. Therefore, based on transcriptome data, DEGs related to phenylpropanoid metabolism and reactive oxygen metabolism were analyzed. Based on the progression of *Rhizopus* rot infection, two critical time points were selected for investigation: the early infection stage (12 h, prior to visible symptom appearance) and active disease progression stage (36 h, when typical rot symptoms became evident).

The expression patterns of phenylpropanoid metabolism-related genes are presented in Figure 6B, highlighting five key enzyme families: PALs, C4Hs, 4CLs, PODs, and PPOs. At 12 h post treatment, E2H treatment induced downregulation of specific genes, including *PpPAL* (LOC18772065 and LOC18784865), *Pp4CL* (LOC18784640, LOC18775963, and LOC18791728), and *PpPOD* (LOC18774150), while upregulating 65% of the detected genes. By 36 h, the treatment universally enhanced gene expression except for *PpPOD* (LOC18774869). This coordinated gene expression profile led to a significant rise in phenylpropanoid metabolism enzyme levels, driving the accumulation of key antimicrobial compounds, particularly total phenolic compounds, ultimately reducing pathogen infection through enhanced biochemical defenses.

The expression profiles of DEGs associated with ROS metabolism are presented in Figure 6C, encompassing three major antioxidant enzyme families: SODs, APXs, and CATs. E2H treatment significantly modulated the expression of 6 *PpSOD* genes, with all members showing upregulation except for *PpMnSOD* (LOC18784827) which was downregulated at 12 h post treatment, and both *PpFeSOD* (LOC18786713) and *PpMnSOD* (LOC18784827), which were downregulated at 36 h post treatment. Similarly, among the four regulated *PpAPX* genes, *PpAPX* (LOC18774632) was downregulated at 12 h and 36 h, *PpAPX2* (LOC18772001) was downregulated at 36 h, and the rest were upregulated. Notably, both *PpCAT* genes (LOC1877304 and LOC18776773) exhibited consistent upregulation throughout the storage period. This comprehensive transcriptional activation resulted in enhanced enzymatic activities, which collectively improved the ROS scavenging capacity, thereby reducing oxidative damage and significantly enhancing resistance to *Rhizopus* rot infection.

## 4. Discussion

E2H, a naturally occurring green leaf volatile compound, demonstrates notable efficacy, safety, and environmental compatibility. As a plant-derived antifungal fumigant, it significantly suppresses phytopathogen proliferation and effectively mitigates postharvest decay in fruit and vegetables by disrupting fungal cell membrane integrity and inhibiting spore germination [21]. Building upon established evidence of the broad-spectrum antiviral activity of E2H and dose-dependent antifungal activity against postharvest pathogens, this study confirmed and extended these findings by demonstrating the optimal efficacy of 2 µL/L E2H in suppressing disease incidence and lesion expansion. Previous research has shown that E2H combated fungi, like *F. graminearum* [46], *Colletotrichum acutatum* [47], and *Penicillium cyclopium* [48], through multimodal mechanisms, including membrane disruption (leading to cellular dysfunction, increased permeability, and cytoplasmic leakage), ROS accumulation, and ultrastructural damage to cell walls and organelles. Consistent with these results, our results indicated that the significant inhibitory effect achieved at 2 µL/L E2H likely stems from similar pathways. These results highlighted the multimodal antifungal action of E2H, which includes membrane disruption, oxidative stress induction, and cellular structural damage, making it a promising candidate for postharvest disease management. Postharvest peach fruit exhibits active physiological metabolism, relying on the breakdown of stored nutrients and energy to maintain cellular functions. Characterized by thin pericarps and soft textures, peaches are particularly prone to rapid ripening and softening after harvest, which accelerates decay and negatively impacts fruit quality, shelf life, and market value [2,3]. E2H, a plant-derived volatile compound, has gained recognition as an eco-friendly and efficient postharvest preservation method due to its ability to delay quality deterioration in various fruits. For instance, E2H treatment has been shown to inhibit browning and softening in kiwifruit while preserving AsA and reducing sugar contents, thereby maintaining sensory quality [21]. In the present study, 2 µL/L E2H treatment effectively mitigated postharvest quality loss in peach fruits by delaying the decline in firmness (Figure 1D) and TSS (Figure 1E), suppressing increases in the yield of juice (Figure 1F) and MDA content (Figure 1G), and enhancing overall storage stability. These findings align with previous reports on E2H’s efficacy in maintaining postharvest quality, further supporting its potential as a sustainable alternative to conventional preservation methods.

PRs are crucial defense proteins induced in plants upon pathogen infection or exogenous elicitor treatment, playing pivotal roles in disease resistance [24]. In this study, CHI and GLU, as two core PRs, effectively inhibited fungal invasion through the specific degradation of key cell wall components (chitin and glucans) [49,50,51]. Total phenolics, the end products of phenylpropanoid metabolism, enhance fruit resistance via dual mechanisms: ROS quenching through phenolic hydroxyl groups, and direct antimicrobial activity. The respiratory burst in postharvest peaches leads to ROS overaccumulation, while total phenolics mitigate oxidative damage by terminating free radical chain reactions and chelating essential metal ions for pathogens. The phenylpropanoid biosynthetic route, critically governed by PAL, C4H, 4CL, POD, and PPO, serves as the core route for total phenolics biosynthesis. The results showed that E2H treatment significantly enhanced the activities of CHI, GLU, and phenylpropanoid enzymes (PAL, C4H, 4CL, POD, PPO). Furthermore, our study utilized RNA-Seq technology to perform differential expression analysis on E2H-treated fruits, revealing that E2H treatment significantly upregulated the expression of phenylpropanoid genes (*PpPAL*, *Pp4CL*, etc.). The results correlated well with the enzymatic analysis.

Correlation analysis revealed strong positive relationships between total phenolics content and phenylpropanoid enzyme activities, demonstrating that increased enzymatic activities drove phenolic accumulation and substantially improved disease resistance. Consistent with previous findings, Li et al. reported that calcium propionate treatment in postharvest pear fruit significantly increased the enzymatic activities and gene expression levels of phenylpropanoid pathway enzymes (PAL, 4CL, C4H, PPO, and POD), significantly boosting the biosynthesis of key defense metabolites like total phenolics, lignin, and flavonoids, leading to increased resistance to *Alternaria alternata* [52]. Caffeic acid treatment was shown to upregulate enzymatic and transcriptional regulation in the phenylpropanoid pathway, significantly elevating biosynthesis of precursor pools, secondary metabolites, structural components, which collectively strengthened disease resistance in pear fruit [53]. 1-Octen-3-ol controlled postharvest brown rot in peaches primarily by upregulating defense-related enzymes (CHI, GLU) as well as activating salicylic acid signaling pathways [54]. Pretreatment with p-coumaric acid and cinnamic acid significantly upregulated PAL, C4H, and 4CL activities in *Agaricus bisporus*, upregulating biosynthesis of defense-associated metabolites like total phenolics, flavonoids, and lignin. These results demonstrated that PCA and CA pretreatment activated the phenylpropanoid pathway to defend against *Pseudomonas tolaasii* infection [55]. Similarly, pathogenesis-responsive genes, like CHI, GLU, and PAL, exhibited significant transcriptional upregulation in peach fruits after treatment with plant glycerol [56], heat treatment [57], and other disease resistance methods, which stimulated the functional capacity of these enzymes within peaches, increased the content of antimicrobial-related substances, and strengthened the resistance of peach fruits to *Rhizopus* rot. These parallel findings underscore the key role of phenylpropanoid metabolism and PRs activation in postharvest disease management in different fruit pathogen systems.

ROS burst represents a characteristic defense response in plants against pathogen infection and mechanical injury [58,59], exhibiting dual physiological effects; while moderate ROS accumulation induces hypersensitive response and programmed cell death (PCD) to promote pathogenesis-related protein synthesis and enhance disease resistance, excessive ROS causes mitochondrial dysfunction and energy metabolism disorder, exacerbating the course of the disease [31]. Previous research has consistently demonstrated the vital importance of antioxidant pathways in enhancing fruit resistance against postharvest pathogens. Wax + octanal treatment markedly elevated the catalytic efficiency of CAT and SOD, thereby enhancing the innate defense capabilities of Satsuma mandarin (*C. unshiu*) to suppress *Penicillium digitatum* infection [60]. Liu et al. identified that *TrPLD1* and *TrPLD2* suppressed the production rate of O_2_^•−^ in apple fruit, while simultaneously activating SOD expression and activity to eliminate excess H_2_O_2_, thereby improving disease resistance [61]. Similarly, Yang et al. investigated the impact of environmental pH on *Fusarium sulphureum* pathogenicity in melon fruit, revealing that at a pH of 3, compared to other pH levels, melon exhibited the highest SOD and CAT activities, the lowest O_2_^•−^ generation rate, and minimal H_2_O_2_ accumulation, resulting in significantly enhanced resistance to fungal infection [62]. Complementary to these findings, chitosan treatment significantly reduced postharvest disease incidence in litchi by enhancing SOD and CAT activities, respectively, suppressing the O_2_^•−^ generation rate and improving DPPH radical scavenging capacity and reducing power, thereby maintaining redox homeostasis and fruit quality during storage [30]. These collective findings underscore the conserved mechanism whereby antioxidant enzyme activation and ROS homeostasis regulation contribute to improving postharvest disease management across diverse fruit species. Our results corroborated these findings, with E2H treatment increasing SOD and CAT activities in peach fruit while effectively inhibiting O_2_^•−^ generation rate and H_2_O_2_ accumulation and preserving both DPPH and hydroxyl radical scavenging capacity. Studies have found that SOD activity was positively correlated with the hydroxyl radical scavenging rate, while SOD activity was negatively correlated with the H_2_O_2_ content and O_2_^•−^ production. Transcriptome analysis revealed E2H-induced upregulation of key ROS metabolic genes (*PpSODs*, *PpAPXs*, and *PpCATs*), consistent with the enzymatic activity measurements. These results collectively demonstrated that E2H treatment orchestrates a multidimensional ROS regulatory network through enhancing antioxidant enzyme activities, suppressing harmful ROS accumulation, and maintaining radical scavenging capacity, ultimately improving peach fruit resistance against *Rhizopus* rot.

As a hub of antioxidative defense, the AsA-GSH cycle coordinates with complementary ROS detoxification networks to preserve redox equilibrium in plant cells. This biochemical cycle operates through coordinated enzymatic reactions: GR reduces GSSG to maintain cellular GSH pools; MDHAR and DHAR utilize GSH to AsA from DHA; APX catalyzes H_2_O_2_ reduction to water at the expense of AsA oxidation, thereby eliminating metabolic free radicals and enhancing disease resistance [63]. Numerous studies have demonstrated that exogenous treatments can effectively activate the AsA-GSH cycle. Acibenzolar-S-methyl (ASM) treatment markedly upregulated the catalytic performance of APX, GR, MDHAR, and DHAR in apple fruit, thereby improving resistance to blue mold (*Penicillium expansum*) [64]. L-cysteine treatment elevated the activities of APX, GR, MDHAR, and DHAR, while promoting the accumulation of reductive substances in plum fruit [65]. Additionally, *Wickerhamomyces anomalus* treatment upregulated the catalytic performance of APX, GR, DHAR, as well as MDHAR in peach fruit, showing that *W. anomalus* stimulated the AsA-GSH cycle, enhanced antioxidant capacity, and consequently improved disease resistance in peaches [3]. Consistent with these findings, our study showed that E2H treatment significantly enhanced AsA-GSH cycle enzyme activities in peach fruit, promoted AsA accumulation, and suppressed DHA production. Correlation analysis revealed strong synergistic interactions among the antioxidant enzymes, collectively maintaining redox balance and improving disease resistance through enhancing ROS scavenging capacity and reducing oxidative damage.

Collectively, our data indicate that E2H treatment suppresses *Rhizopus* rot in peach fruit by reducing disease incidence and lesion diameter while enhancing postharvest storage quality. This resistance is mediated through PR protein activation (CHI and GLU), phenylpropanoid metabolism upregulation, and ROS homeostasis modulation.

## 5. Conclusions

Our findings established that E2H treatment markedly suppressed *Rhizopus* rot progression in peach fruit, as evidenced by reduced disease incidence and restricted lesion expansion, while concurrently preserving postharvest quality. The treatment elevated activities of PR proteins (CHI and GLU), while enhancing key phenylpropanoid pathway enzymes including PAL, C4H, 4CL, POD, and PPO, thereby promoting total phenolic accumulation. Concurrently, E2H treatment increased SOD and CAT activities, suppressed H_2_O_2_ accumulation, and maintained high DPPH and hydroxyl radical scavenging capacities. Furthermore, it activated the AsA-GSH cycle by elevating MDHAR, DHAR, APX, as well as GR activities, resulting in increased AsA content and decreased DHA production. These coordinated biochemical responses collectively enhanced the antioxidant defense system and improved resistance against *Rhizopus* rot infection in peach fruit. As a result, we recommend 2 µL/L E2H fumigation as an eco-friendly alternative to chemical fungicides. However, the molecular mechanisms underlying these metabolic pathways, particularly the signaling networks and gene regulatory elements involved, warrant further investigation to fully elucidate their contributions to E2H-mediated disease resistance.

## Data Availability

The original contributions presented in the study are included in the article, further inquiries can be directed to the corresponding author.

## Abbreviations

The following abbreviations are used in this manuscript:

E2H	Trans-2-hexenal
MDA	Malondialdehyde
PAL	Phenylalanine ammonia-lyase
C4H	Cinnamate 4-hydroxylase
4CL	4-coumarate-CoA ligase
PPO	Polyphenol oxidase
POD	Peroxidase
CHI	Chitinase
GLU	β-1,3-glucanase
AsA	Ascorbic acid
DHA	Dehydroascorbic acid
ROS	Reactive oxygen species
PR	Pathogenesis-related protein
HR	Hypersensitive response
PCD	Programmed cell death
SOD	Superoxide dismutase
CAT	Catalase
APX	Ascorbate peroxidase
GR	Glutathione reductase
GSH	Glutathione
GABA	γ-aminobutyric acid
PDA	Potato dextrose agar
TSS	Total soluble solids
N	Newtons
TBA	Thiobarbituric acid method
TCA	Trichloroacetic acid
DMAB	P-dimethylaminobenzaldehyde
•OH	Hydroxyl radical
O_2_^•−^	Superoxide anion
H_2_O_2_	Hydrogen peroxide
MDHAR	Monodehydroascorbate reductase
DHAR	Dehydroascorbate reductase
GLM	generalized linear model
FDR	False discovery rate
Fig.	Figure
TPC	Total phenolic content
•OH-SR	hydroxyl radical scavenging rate
ASM	Acibenzolar-S-methyl

## References

[1] Wang L., Shan T.M., Xie B., Ling C., Shao S., Jin P., Zheng Y.H. (2019). Glycine betaine reduces chilling injury in peach fruit by enhancing phenolic and sugar metabolisms. Food Chem..

[2] Jiang J.Y., Gong L., Dong Q.F., Kang Y.F., Osako K., Li L. (2020). Characterization of PLA-P3,4HB active film incorporated with essential oil: Application in peach preservation. Food Chem..

[3] Zhou Y.L., Zhao L., Chen Y.Q., Dhanasekaran S., Chen X.F., Zhang X.Y., Yang X.Z., Wu M.Y., Song Y.D., Zhang H.Y. (2024). Study on the control effect and physiological mechanism of *Wickerhamomyces anomalus* on primary postharvest diseases of peach fruit. Int. J. Food Microbiol..

[4] Mustafa M.H., Bassi D., Corre M.N., Lino L.O., Signoret V., Quilot-Turion B., Cirilli M. (2021). Phenotyping brown rot susceptibility in stone fruit: A literature review with emphasis on peach. Horticulturae.

[5] Shi Y.Y., Zhang S., Zhang J.H., Wang X.R., He Y., Han X.D., Song Z.Y., Shi J.Y. (2024). The volatile components from *Bacillus cereus* N4 can restrain brown rot of peach fruit by inhibiting sporulation of *Monilinia fructicola* and inducing disease resistance. Postharvest Biol. Technol..

[6] Angeli S.S., De Mio L.L.M., Amorim L. (2017). Comparative analysis of *Monilinia fructicola* and M-laxa isolates from Brazil: Monocyclic components of peach brown rot. Cienc. Rural..

[7] Romanazzi G., Sanzani S.M., Bi Y., Tian S.P., Martínez P.G., Alkan N. (2016). Induced resistance to control postharvest decay of fruit and vegetables. Postharvest Biol. Technol..

[8] Dai B.E., Wang Y.X., Zhou H.J., Wang L.F., Zhou L., Mao J.X., Zhang S.Y., Shen S.L., Zheng X.L., Huan C. (2024). Control efficiency and potential mechanisms of chlorogenic acid against postharvest gray mold caused by *Botrytis cinerea* on peach fruit. Postharvest Biol. Technol..

[9] Wang X.L., Xu F., Wang J., Jin P., Zheng Y.H. (2013). *Bacillus cereus* AR156 induces resistance against *Rhizopus* rot through priming of defense responses in peach fruit. Food Chem..

[10] Xu B.T., Zhang H.Y., Chen K.P., Xu Q., Yao Y., Gao H. (2013). Biocontrol of postharvest *Rhizopus* decay of peaches with *Pichia caribbica*. Curr. Microbiol..

[11] Adnan M., Hamada M.S., Hahn M., Li G.Q., Luo C.X. (2019). Fungicide resistance of *Botrytis cinerea* from strawberry to procymidone and zoxamide in Hubei, China. Phytopathol. Res..

[12] Mou L.Y., Du X.L., Lu X.F., Lu Y., Li G.P., Li J.L. (2021). Component analysis and antifungal activity of three Chinese herbal essential oils and their application of postharvest preservation of peach fruit. LWT-Food Sci. Technol..

[13] Gura W.P., Gelain J., Sikora E.J., Vinson E.L., Brannen P.M., Schnabel G. (2023). Low frequency of resistance to thiophanate-methyl in *Monilinia fructicola* populations from southeastern United States peach orchards. Pestic. Biochem. Physiol..

[14] Saito S., Michailides T.J., Xiao C.L. (2016). Fungicide resistance profiling in *Botrytis cinerea* populations from blueberry in California and Washington and their impact on control of gray mold. Plant Dis..

[15] Kaur N., Shahwar D., Hassan F.E., Ahmed Z.F.R. (2023). Antioxidant and antibacterial activities of date palm fruit (*Phoenix dactylifera* L.) in response to postharvest application with natural elicitors. Acta Hortic..

[16] OuYang Q.L., Okwong R.O., Chen Y.P., Tao N.G. (2020). Synergistic activity of cinnamaldehyde and citronellal against green mold in citrus fruit. Postharvest Biol. Technol..

[17] Zhou D.D., Wang B., Chen W., Wang Y.B., Sun Y.Y., Zhang M.Y., He J. (2025). Mushroom alcohol treatment inhibited the growth of *Alternaria tenuissima* and reduced the incidence of postharvest diseases of fresh wolfberry fruit. Food Control..

[18] Guo M.R., Feng J.Z., Zhang P.Y., Jia L.Y., Chen K.S. (2015). Postharvest treatment with trans-2-hexenal induced resistance against *Botrytis cinerea* in tomato fruit. Australas. Plant Pathol..

[19] Hyun J., Lee J.G., Yang K.Y., Lim S., Lee E.J. (2022). Postharvest fumigation of (E)-2-hexenal on kiwifruit (*Actinidia chinensis* cv. ‘Haegeum’) enhances resistance to *Botrytis cinerea*. Postharvest Biol. Technol..

[20] Ma W.B., Zhao L.L., Xie Y.L. (2017). Inhibitory effect of (E)-2-hexenal as a potential natural fumigant on *Aspergillus flavus* in stored peanut seeds. Ind. Crop. Prod..

[21] Yuan X.X., Meng K.X., Shi S.W., Wu Y.Y.B., Chen X.M., OuYang Q.L., Tao N.G. (2023). Trans-2-hexenal inhibits the growth of imazalil-resistant *Penicillium digitatum* Pdw03 and delays green mold in postharvest citrus. Postharvest Biol. Technol..

[22] Di Francesco A., Martini C., Mari M. (2016). Biological control of postharvest diseases by microbial antagonists: How many mechanisms of action?. Eur. J. Plant Pathol..

[23] Sels J., Mathys J., De Coninck B.M.A., Cammue B.P.A., De Bolle M.F.C. (2008). Plant pathogenesis-related (PR) proteins: A focus on PR peptides. Plant Physiol. Biochem..

[24] Abeles F.B., Bosshart R.P., Forrence L.E., Habig W.H. (1971). Preparation and purification of glucanase and chitinase from bean leaves. Plant Physiol..

[25] Ge Y.H., Duan B., Li C.Y., Tang Q., Li X., Wei M.L., Chen Y.R., Li J.R. (2018). γ-Aminobutyric acid delays senescence of blueberry fruit by regulation of reactive oxygen species metabolism and phenylpropanoid pathway. Sci. Hortic..

[26] Guo Y., Li X., Li C.Y., Jinyue R.X., Xu H.P., Ge Y.H. (2023). Acibenzolar-S-methyl activates phenylpropanoid pathway to enhance resistance against *Alternaria alternata* in pear fruit. J. Sci. Food Agric..

[27] Li Q., Wei Y.Y., Chen Y., Jiang S., Ye J.F., Xu F., Lou Y.J., Ding P.B., Ouaziz M., Shao X.F. (2024). Agaro-oligosaccharides enhanced the *Monilinia fructicola* resistance of peach fruit by regulating antioxidative and phenylpropanoid metabolism. Postharvest Biol. Technol..

[28] Xie G.F., Liu N., Zhang Y., Tan S.M., Xu Y.Q., Luo Z.S. (2024). Postharvest MeJA maintains the shelf quality of kiwifruit after cold storage by regulating antioxidant capacity and activating the disease resistance. Postharvest Biol. Technol..

[29] Zhang X., Wang L., Chen Y.Y., Dai Y., Li M.Q., Zhang H.W. (2025). Natamycin and potassium sorbate synergistically enhance resistance to *Botrytis cinerea* by activating the phenylpropanoid metabolism in harvested strawberry. Postharvest Biol. Technol..

[30] Jiang X.J., Lin H.T., Lin M.S., Chen Y.H., Wang H., Lin Y.X., Shi J., Lin Y.F. (2018). A novel chitosan formulation treatment induces disease resistance of harvested litchi fruit to *Peronophythora litchii* in association with ROS metabolism. Food Chem..

[31] Zhao Y.N., Yu H., Zhou J.M., Smith S.M., Li J.Y. (2020). Malate circulation: Linking chloroplast metabolism to mitochondrial ROS. Trends Plant Sci..

[32] Yang W.T., Li L.L., Liu Y.X., Zhang W.D., Guo M.R., Chen G.G. (2025). MeJA and MeSA alleviate black rot in winter jujube caused by *Alternaria tenuissima* by regulating membrane lipid and reactive oxygen metabolism. Postharvest Biol. Technol..

[33] Ding X.C., Liu S., Duan X.W., Pan X.J., Dong B.Y. (2023). MAPK cascade and ROS metabolism are involved in GABA-induced disease resistance in red pitaya fruit. Postharvest Biol. Technol..

[34] Wang X.L., Zhu J.F., Wei H., Ding Z.P., Li X.R., Liu Z., Wang H.B., Wang Y.P. (2023). Biological control efficacy of *Bacillus licheniformis* HG03 against soft rot disease of postharvest peach. Food Control..

[35] Shan T.M., Jin P., Zhang Y., Huang Y.P., Wang X.L., Zheng Y.H. (2016). Exogenous glycine betaine treatment enhances chilling tolerance of peach fruit during cold storage. Postharvest Biol. Technol..

[36] Li D., Limwachiranon J., Li L., Du R.X., Luo Z.S. (2016). Involvement of energy metabolism to chilling tolerance induced by hydrogen sulfide in cold-stored banana fruit. Food Chem..

[37] Zhang M.Y., Wang D.J., Gao X.X., Yue Z.Y., Zhou H.L. (2020). Exogenous caffeic acid and epicatechin enhance resistance against *Botrytis cinerea* through activation of the phenylpropanoid pathway in apples. Sci. Hortic..

[38] Zhu L.J., Yu H.T., Dai X.M., Yu M.L., Yu Z.F. (2022). Effect of methyl jasmonate on the quality and antioxidant capacity by modulating ascorbate-glutathione cycle in peach fruit. Sci. Hortic..

[39] Hou Y.Y., Li Z.Y., Zheng Y.H., Jin P. (2021). Effects of CaCl_2_ Treatment Alleviates Chilling Injury of Loquat Fruit (*Eribotrya japonica*) by Modulating ROS Homeostasis. Foods.

[40] Zuo X.X., Cao S.F., Jia W.R., Zhao Z.Y., Jin P., Zheng Y.H. (2021). Near-saturated relative humidity alleviates chilling injury in zucchini fruit through its regulation of antioxidant response and energy metabolism. Food Chem..

[41] Alexieva V., Sergiev I., Mapelli S., Karanov E. (2001). The effect of drought and ultraviolet radiation on growth and stress markers in pea and wheat. Plant Cell Environ..

[42] Ma Y.Q., Hu S.Q., Chen G.F., Zheng Y.H., Jin P. (2022). Cold shock treatment alleviates chilling injury in peach fruit by regulating antioxidant capacity and membrane lipid metabolism. Food Qual. Saf..

[43] Zhang Y., Jin P., Huang Y.P., Shan T.M., Wang L., Li Y.Y., Zheng Y.H. (2016). Effect of hot water combined with glycine betaine alleviates chilling injury in cold-stored loquat fruit. Postharvest Biol. Technol..

[44] Chumyam A., Shank L., Faiyue B., Uthaibutra J., Saengnil K. (2017). Effects of chlorine dioxide fumigation on redox balancing potential of antioxidative ascorbate-glutathione cycle in ‘Daw’ longan fruit during storage. Sci. Hortic..

[45] Wang Q., Ding T., Zuo J.H., Gao L.P., Fan L.L. (2016). Amelioration of postharvest chilling injury in sweet pepper by glycine betaine. Postharvest Biol. Technol..

[46] Ma D.C., Wang G.X., Zhu J.M., Mu W., Dou D.L., Liu F. (2022). Green leaf volatile trans-2-hexenal inhibits the growth of *Fusarium graminearum* by inducing membrane damage, ROS accumulation, and cell dysfunction. J. Agric. Food Chem..

[47] Arroyo F.T., Moreno J., Daza P., Boianova L., Romero F. (2007). Antifungal activity of strawberry fruit volatile compounds against *Colletotrichum acutatum*. J. Agric. Food Chem..

[48] Zhang J.H., Tian H., Sun H.L., Wang X.Y. (2017). Antifungal activity of trans-2-hexenal against *Penicillium cyclopium* by a membrane damage mechanism. J. Food Biochem..

[49] Curto M.A., Butassi E., Ribas J.C., Svetaz L.A., Cortés J.C.G. (2021). Natural products targeting the synthesis of β(1,3)-D-glucan and chitin of the fungal cell wall. Existing drugs and recent findings. Phytomedicine.

[50] Tzelepis G., Karlsson M. (2019). Killer toxin-like chitinases in filamentous fungi: Structure, regulation and potential roles in fungal biology. Fungal Biol. Rev..

[51] Ma J.W., Qin Z., Zhou P., Wang R.M., Yan Q.J., Jiang Z.Q., Yang S.Q. (2022). Structural insights into the substrate recognition and catalytic mechanism of a fungal glycoside hydrolase family 81 β-1,3-glucanase. Enzym. Microb. Technol..

[52] Li C.Y., Wang M., Guo Y., Zhang S.R., Xu H.P., Ge Y.H. (2024). Activation of the calcium signaling, mitogen-activated protein kinase cascade and phenylpropanoid metabolism contributes to the induction of disease resistance in pear fruit upon phenylalanine treatment. Postharvest Biol. Technol..

[53] Guo Y., Li C.Y., Wang M., Xu H.P., Zhang S.R., Liu J.Q., Jin Y.R.X., Ge Y.H. (2024). Postharvest caffeic acid dipping enhances disease resistance and storage capacity of ‘Zaosu’ pear fruit via regulating phenylpropanoid metabolism. Postharvest Biol. Technol..

[54] Wang X.Z., Huang M.M., Peng Y., Yang W.T., Shi J.Y. (2022). Antifungal activity of 1-octen-3-ol against *Monilinia fructicola* and its ability in enhancing disease resistance of peach fruit. Food Control.

[55] Shi Z.X., Song R., Zhang L., Jiang H.Y., Jiao L., Yuan S., Zheng Y.Y., Chen L., Meng D.M. (2025). Para-coumaric acid and cinnamic acid enhance resistance of *Agaricus bisporus* s mushrooms to Brown blotch disease caused by *Pseudomonas tolaasii*. Food Control.

[56] Zhang Q.C., Li W.H., Han X.D., Wu B., Song Z.Y., Shi J.Y. (2024). Plant glycerol suppresses brown rot of peach fruit by enhancing disease resistance. Physiol. Mol. Plant Pathol..

[57] Liu J., Sui Y., Wisniewski M., Droby S., Tian S.P., Norelli J., Hershkovitz V. (2012). Effect of heat treatment on inhibition of *Monilinia fructicola* and induction of disease resistance in peach fruit. Postharvest Biol. Technol..

[58] Wang Y., Ji D.C., Chen T., Li B.Q., Zhang Z.Q., Qin G.Z., Tian S.P. (2019). Production, signaling, and scavenging mechanisms of reactive oxygen species in fruit-pathogen interactions. Int. J. Mol. Sci..

[59] Zhang Z.Q., Chen Y., Li B.Q., Chen T., Tian S.P. (2020). Reactive oxygen species: A generalist in regulating development and pathogenicity of phytopathogenic fungi. Comput. Struct. Biotechnol. J..

[60] Tao N.G., Fan F., Jia L., Zhang M.L. (2014). Octanal incorporated in postharvest wax of Satsuma mandarin fruit as a botanical fungicide against *Penicillium digitatum*. Food Control.

[61] Liu Q.L., Zhang Q.Q., Xue H.L., Bi Y., Yang X., Zong Y.Y., Liu Z.G., Chen J.Y., Prusky D. (2023). *TrPLD1* and *TrPLD2* modulate reactive oxygen species production and pathogenicity in *Trichothecium roseum* infected apple fruit. Postharvest Biol. Technol..

[62] Yang L., Xue H.L., Liu Z.G., Liu Q.L., Zhang Q.Q., Nan M.N. (2022). The effects of different ambient pH on the pathogenicity of *Fusarium sulphureum* and reactive oxygen species metabolism in *F. sulphureum* inoculation muskmelon fruits. Physiol. Mol. Plant Pathol..

[63] Ma Y.Y., Huang D.D., Chen C.B., Zhu S.H., Gao J.G. (2019). Regulation of ascorbate-glutathione cycle in peaches via nitric oxide treatment during cold storage. Sci. Hortic..

[64] Wei M.L., Ge Y.H., Li C.Y., Han X., Qin S.C., Chen Y.R., Tang Q., Li J.R. (2019). G6PDH regulated NADPH production and reactive oxygen species metabolism to enhance disease resistance against blue mold in apple fruit by acibenzolar-S-methyl. Postharvest Biol. Technol..

[65] Wang W.J., Ling Y., Deng L.L., Yao S.X., Zeng K.F. (2023). Effect of L-cysteine treatment to induce postharvest disease resistance of *Monilinia fructicola* in plum fruits and the possible mechanisms involved. Pestic. Biochem. Physiol..

